# Successful transplantation of guinea pig gut microbiota in mice and its effect on pneumonic plague sensitivity

**DOI:** 10.7717/peerj.5637

**Published:** 2018-09-24

**Authors:** Xiang Li, Zhengchao Li, Yuxiao Chang, Fengyi Hou, Zongyu Huang, Han Ni, Ruifu Yang, Yujing Bi

**Affiliations:** State Key Laboratory of Pathogen and Biosecurity, Beijing Institute of Microbiology and Epidemiology, Beijing, China

**Keywords:** Transplantation, Gut microbiota, Guinea pig, Mice, *Yersinia pestis*

## Abstract

Microbiota-driven variations in the inflammatory response are predicted to regulate host responses to infection. Increasing evidence indicates that the gastrointestinal and respiratory tracts have an intimate relationship with each other. Gut microbiota can influence lung immunity whereby gut-derived injurious factors can reach the lungs and systemic circulation via the intestinal lymphatics. The intestinal microbiota’s ability to resist colonization can be extended to systemic infections or to pathogens infecting distant sites such as the lungs. Unlike the situation with large mammals, the microtus *Yersinia pestis* 201 strain exhibits strong virulence in mice, but nearly no virulence to large mammals (such as guinea pigs). Hence, to assess whether the intestinal microbiota from guinea pigs was able to affect the sensitivity of mice to challenge infection with the *Y. pestis* 201 strain, we fed mice with guinea pig diets for two months, after which they were administered 0.5 ml of guinea pig fecal suspension for 30 days by oral gavage. The stools from each mouse were collected on days 0, 15, and 30, DNA was extracted from them, and 16S rRNA sequencing was performed to assess the diversity and composition of the gut microbiota. We found that the intestinal microbiota transplants from the guinea pigs were able to colonize the mouse intestines. The mice were then infected with *Yersinia pestis* 201 by lung invasion, but no statistical difference was found in the survival rates of the mice that were colonized with the guinea pig’s gut microbiota and the control mice. This indicates that the intestinal microbiota transplantation from the guinea pigs did not affect the sensitivity of the mice to pneumonic plague.

## Introduction

The human intestine is inhabited by nearly 100 trillion microorganisms, including more than 1,000 distinct bacterial species (Feng & Elson, 2011), and these microorganisms are referred to as the intestinal microbiota. Microbiota-driven variations in the inflammatory response are predicted to regulate the host response to infection (Blander et al., 2017). The microbiota prevents pathogen invasion by competing with them for space and nutrients, secreting antimicrobial peptides, stimulating innate immune cells, promoting lymphoid tissue development and T cell differentiation, and producing antibodies (Belkaid & Naik, 2013; Blander et al., 2017; Kamada et al., 2013).

The microbiome not only modulates intestinal tract immunity, but operates on distal sites as well (Kieper et al., 2005). Many studies have shown that having healthy gut bacteria is beneficial to lung health. A recent report indicates that the gut microbiome has the ability to regulate lung inflammation, as a diverse range of intestinal microbiota helps to maintain lung homeostasis by supporting immune function in this organ, while intestinal microbiota dysbiosis enhances lung inflammation in response to allergens or infections (McAleer & Kolls, 2018). The gut and lung microbiota are shown to communicate with each other, so that when the composition or function of the microbiota changes in either organ, it can affect the other. This interaction between the gut and lungs has been termed the gut-lung axis (Chakradhar, 2017), and an increasing amount of evidence supports its existence (Chen et al., 2017). For example, during pneumococcal pneumonia, the intestinal microbiota plays a protective role. When wild-type mice had their gut microbiota depleted by drinking broad-spectrum antibiotics and were subsequently infected with *Streptococcus pneumoniae*, the causative agent of pneumococcal pneumonia, the mice showed an accelerated mortality rate and an increased bacterial load in the lungs at 6 h post-infection. Moreover, the capacity of the alveolar macrophages was diminished when compared with mice that did not receive antibiotics (Schuijt et al., 2016). In germ-free mice, the lack of gut microbiota enhanced susceptibility to *Klebsiella pneumonia*, and led to an interleukin-10-mediated inflammatory state (Fagundes et al., 2012). In addition to its role in protecting from bacterial infection, the intestinal microbiota also plays a crucial role in the immune response to respiratory viral infections. The innate and adaptive immune responses in antibiotic-treated mice were defective against the influenza virus, and the loss of immunoregulation was related to virus-specific CD4 and CD8 T cell subsets (Chen et al., 2017). Stimulation of immune cells by microbial cells and their associated metabolites at one site can affect the cells at another site; hence, changes in the gut microbiome can influence lung immunity. The microbiota in the gut can influence lung immunity, and gut-derived tissue-injurious factors can reach this organ and the systemic circulation via the intestinal lymphatic system. Thus, the gut microbiota can affect the gut-lung axis host defenses and promote pulmonary disease.

*Yersinia pestis*, the agent of plague, has historically been divided into the antiqua, mediaevalis, and orientalis biovars (Perry & Fetherston, 1997). In 2004, a fourth biovar was identified and sequenced, and named microtus (Song et al., 2004). *Y. pestis* 201, a microtus strain of *Y. pestis*, displays strong virulence in mice but not in larger mammals (Fan et al., 1995). The biovar assignment for this strain is based on metabolic variations that do not seem to correlate with its virulence characteristics. Likewise, the difference in its genetic makeup does not explain its virulence profile. Recently, findings were published from a study conducted around 40 years ago on the sensitivity of *Meriones* rodents to plague in Iran (Assmar et al., 2018). The authors found that plague resistance could be transmitted from plague-resistant *Meriones* to their plague-sensitive counterparts by fecal transplantation. However, at the time the study was conducted, the importance of the role of gut microbiota in health and disease was not known. Evidence from several studies thus indicates a possible relationship between gut-lung axis and plague susceptibility. Since larger mammals, including guinea pigs, show resistance to *Y. pestis* (201 strain) infection, here we transplanted the intestinal microbiota of guinea pigs to pneumonic plague-sensitive mice to investigate whether guinea pig gut microbiota affects mouse susceptibility to *Y. pestis* infection.

## Materials and Methods

Female BALB/C mice (6–8 weeks of age) and guinea pigs (4 weeks of age) were purchased from Beijing Vital River Laboratory Animal Technology Co. Ltd. (Beijing, China). All animals were maintained under specific pathogen-free controlled conditions (22 °C, 55% humidity, 12 h/12 h dark/light cycle). The mouse normal chow diets and the guinea pig normal chow diets were sourced from the Experimental Animal Center, Beijing, China. All food was irradiated and drinking water was autoclaved. This study was approved by the Institutional Animal Care and Use Committee: Medical Ethical Committee, Academy of Military Medical Sciences (No.SCXK-2016-01070126).

The *Y. pestis* strain used in this study was the wild-type 201 strain isolated from *Microtus brandti* in Inner Mongolia, China, which belongs to the newly established *Y. pestis* microtus biovar (Zhou et al., 2004). This 201 strain is lethal in mice, but nearly avirulent in humans (Fan et al., 1995).

### Pretreatment

Twenty mice were randomly divided into two groups (*n* = 10), one of which was fed a guinea pig diet (experimental group) and the other fed a mouse diet (control group), both for two months.

### Fecal microbial transplantation (FMT)

Guinea pig stool suspensions were prepared as follows. Three guinea pig stool samples were collected, 9 mL of phosphate-buffered saline was added, and the samples were mixed well to make a suspension. The experimental group received 0.5 mL of the guinea pig stool suspension by gavage daily for 30 days, and were fed a guinea pig diet throughout this period. The control group received 0.5 mL of a mouse stool suspension by gavage daily for 30 days, and were fed a mouse diet throughout this period. Stool samples from these two groups were collected on days 0, 15 and 30.

### DNA extraction

Bacterial DNA from the stool samples was extracted using the QIAamp DNA Stool Mini Kit (QIAGEN, Hilden, Germany) according to the manufacturer’s protocol. The extracted bacterial DNA was stored at −80 °C until sequence analysis.

### 16S rRNA gene sequencing

The V4 region of the 16S rRNA gene was selected as the sequencing region with which to compare the diversity and structure of the bacterial species in each of the samples. The primers for the V4 region were 515F (GTGCCAGCMGCCGCGGTAA) and 806R (GGACTACHVGGGTWTCTAAT). Sequencing was performed by Illumina MiSeq at Novogene Bioinformatics Technology Co., Ltd. (Beijing, China).

### Sequence analysis

### Processing of raw data

Raw sequencing data was joined and spliced into a single sequence. Individual sequences from each sample were distinguished according to their barcode sequences. Low quality sequences and sequences that could not be compared with the 16S rRNA database were filtered out.

### Operational taxonomic unit (OTU) classification and statistics

16S rRNA sequence data was analyzed using Uparse software (Uparse v7.0.1001, http://drive5.com/uparse/), which divided the sequences into OTUs using a similarity threshold of 97%. A similarity score of <97% was considered indicative of a different species, and a score of <93%–95% was considered indicative of a different genus. The SSUrRNA database was used to annotate the species, and MUSCLE (Version 3.8.31, http://www.drive5.com/muscle/) was used to BLAST the sequences. Finally, all data were normalized for further analysis.

### Analysis of *α*- and *β*-diversity

Diversity within a sample (α-diversity) and between samples (β-diversity) was estimated on the basis of the OTUs. α-diversity refers to the diversity of a particular region or ecosystem and is an expression of the species diversity in a single sample. α-diversity includes community richness (as defined by the Chao and ACE indices) and community diversity (as defined by the Shannon index). β-diversity indices were used to estimate the distance between samples based on the evolutionary relationship of the sample’s sequence and its abundance. β-diversity indices are expressed in terms of the differences between the sample groups by means of principal coordinate analysis (PCoA).

### Linear discriminant analysis (LDA)

Metastat and LDA effect size (LefSe) analysis were used to detect significant differences among groups in the microbial communities’ biomarkers.

### *Y. pestis* infections and histopathology observation

*Y. pestis* was grown overnight at 26 °C with continuous shaking in Bacto heart infusion broth (BD, USA). After dilution to an optical density of 0.1 at 620 nm, the bacteria were grown continuously overnight at 26 °C, collected by centrifugation, washed with saline, and then quantified by optical density measurement and adjusted to the desired concentration. The experimental group (guinea pig diet and guinea pig stool gavage) and control group (mouse diet and mouse stool gavage) were randomly subdivided into a low dose group and a high dose group. At the end of the 30-day fecal microbial transplantation period, the *Y. pestis* suspension was injected into the lungs through a laryngoscope. The number of bacteria in the inoculating dose was confirmed by plating experiments. The low dose group was injected with two colony forming units (CFU) of bacteria, and the high dose group was injected with fifteen CFU of bacteria.

Immediately after the mice were euthanized, the lungs were dissected out and placed into 10% neutral buffered formalin, dehydrated through a serial alcohol gradient (70%, 80%, 90%, 95%, and 100%), cleared with xylene, infiltrated with wax, and then embedded in paraffin. Tissue sections were stained with hematoxylin and eosin for histopathological examination.

## Results

**Figure 1:**
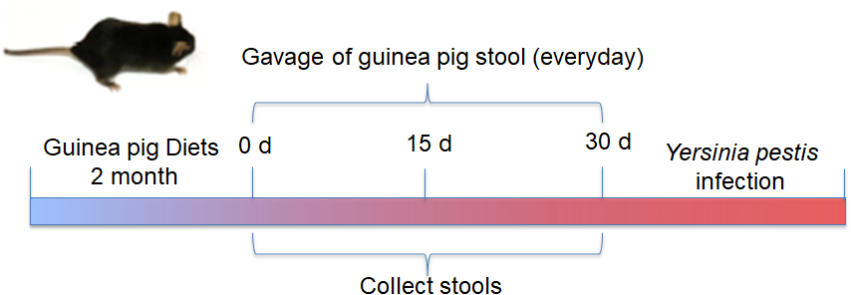
Schematic diagram showing the design and timeline of the mice experiments. Female BALB/C mice (6–8 weeks old) were either fed a guinea pig diet for 2 months and then given 0.5 mL of guinea pig stool suspension by oral gavage daily for 30 days (experimental group), or fed a mouse diet for 2 months and then given 0.5 mL of mouse stool suspension by oral gavage daily for 30 days (control group). Stool samples from each mouse were collected on days 0, 15, and 30. Stool samples from the experimental group were marked T0d, T15d and T30d, and those from the control group were marked S0d, S15d and S30d (*n* = 9–10 mice per group). After the 30-day fecal microbial transplantation (FMT) period, experimental and control mice were infected intranasally with *Yersinia pestis* for 15 days.

To determine whether gut microbiota from guinea pig stool samples could colonize the mouse gut, we began by feeding mice on a guinea pig diet for two months, then administered a guinea pig stool suspension by gavage each day for 30 days (Fig. 1). Control group mice were fed a standard mouse diet, then received a mouse stool suspension for their FMT. Stool samples were collected on days 0, 15 and 30 as indicated in Fig. 1. Stool samples from the experimental group were marked T0d, T15d and T30d, those from the control group were marked S0d, S15d and S30d, and those from guinea pigs were marked Tcon. DNA extracted from the stool samples was sequenced by 16s rRNA sequencing.

**Figure 2:**
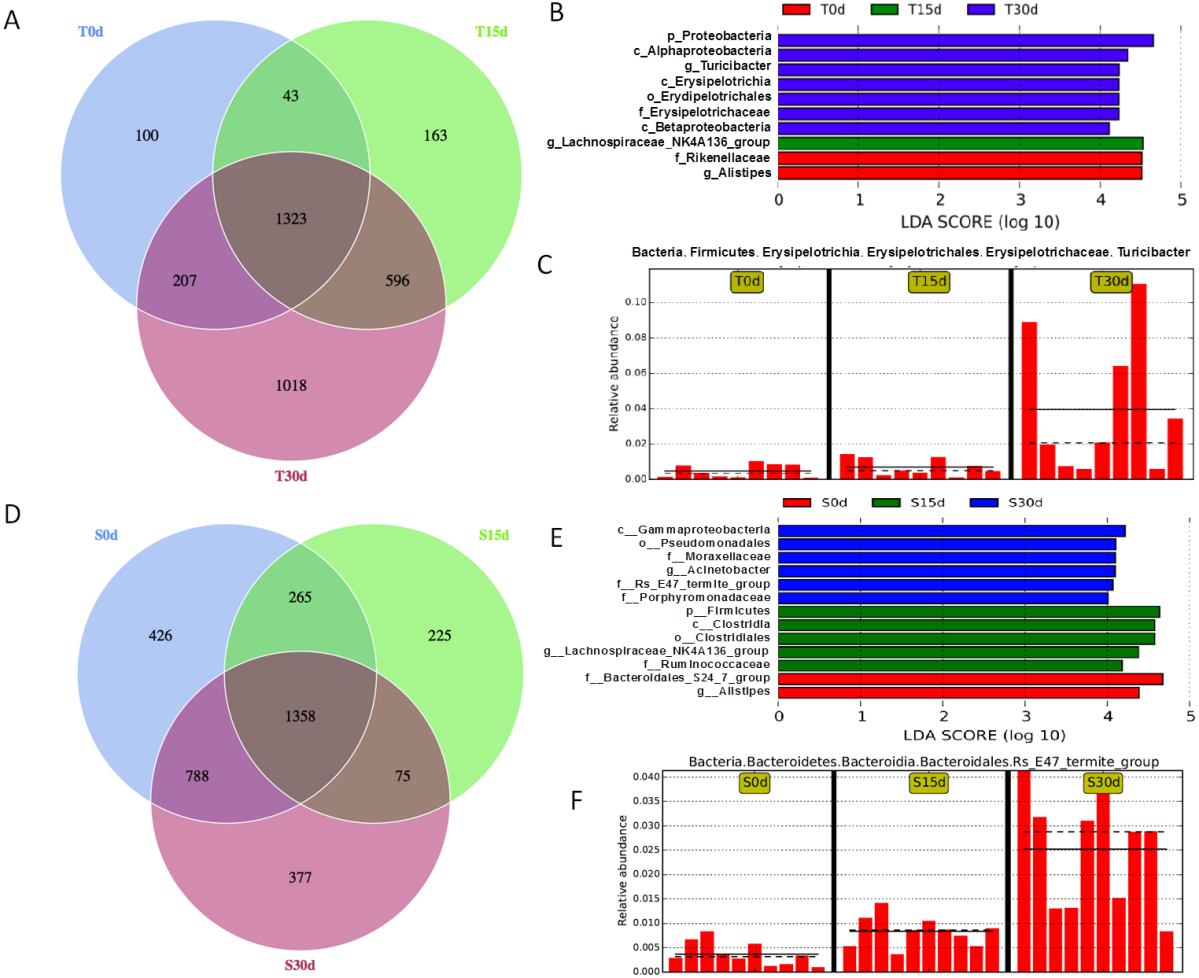
During FMT the gut microbiota regulation. Venn diagrams highlighting the unique and shared OTUs in the different communities with (A) representing the T0d, T15d, and T30d communities, and (D) representing the S0d, S15d, and T30d communities. The LefSe value distribution histogram shows the species where the LDA value exceeds the given value. The significantly enriched bacterial taxa in the communities from the experimental group (B) and from the control group (E) as determined by LEfSe analysis, where the differences are represented by the color of the most abundant class. The length of the histogram represents the LDA score, that is, the extent of the significant difference among the different groups. (C and F) Relative abundance histogram. The solid line is the relative abundance average. The dashed line is the median relative abundance. Each column represents the relative abundance of each sample in each group (*n* = 9–10 mice per group).

To compare the changes in the gut microbiota during the 30-day FMT between the experimental (guinea pig stool FMT) group and control (mouse stool FMT) group, a Venn diagram was used. In the experimental group, 1323 OTUs were common to all time point samples, and 100, 163 and 1018 OTUs were identified in T0d, T15d and T30d samples only, respectively (Fig. 2A). In the control group, 1358 OTUs were common to all time point samples, and 462, 225 and 377 OTUs were identified in S0d, S15d and S30d samples only, respectively (Fig. 2D). The high number of OTUs specific to T30d suggests that the guinea pig stool FMT changed the composition of the mouse gut microbiota.

To identify the specific bacterial taxa associated with the guinea pig stool FMT, we analyzed the composition of the gut microbiota of the experimental and control groups separately using the LEfSe method. In the experimental group, the LEfSe analysis revealed 10 discriminative features (LDA > 4, *P* < 0.05, Fig. 2B). Two taxa (Rikenellaceae and Alistipes) showed increased abundance in T0d, one taxon (Lachnospiraceae NK4A136 group) showed increased abundance in T15d, and several taxa showed increased abundance in T30d (e.g., Proteobacteria, Alphaproteobacteria, Turicibacter, Erysipelotrichaceae and Betaproteobacteria). A typical bacterial profile for the experimental group is shown in Fig. 2C, and other changes are shown in Fig. S1. In the control group, 13 discriminative features were found by LEfSe analysis (LDA > 4, *P* < 0.05, Fig. 2E). A typical bacterial profile for the control group is shown in Fig. 2F, and other changes are shown in Fig. S2. Both the experimental and control groups showed an increase in bacterial abundance with time; however, the identities of these bacteria at day 30 differ between these two groups, indicative of a difference in the nature of the change in gut microbiota over time between the two groups.

**Figure 3:**
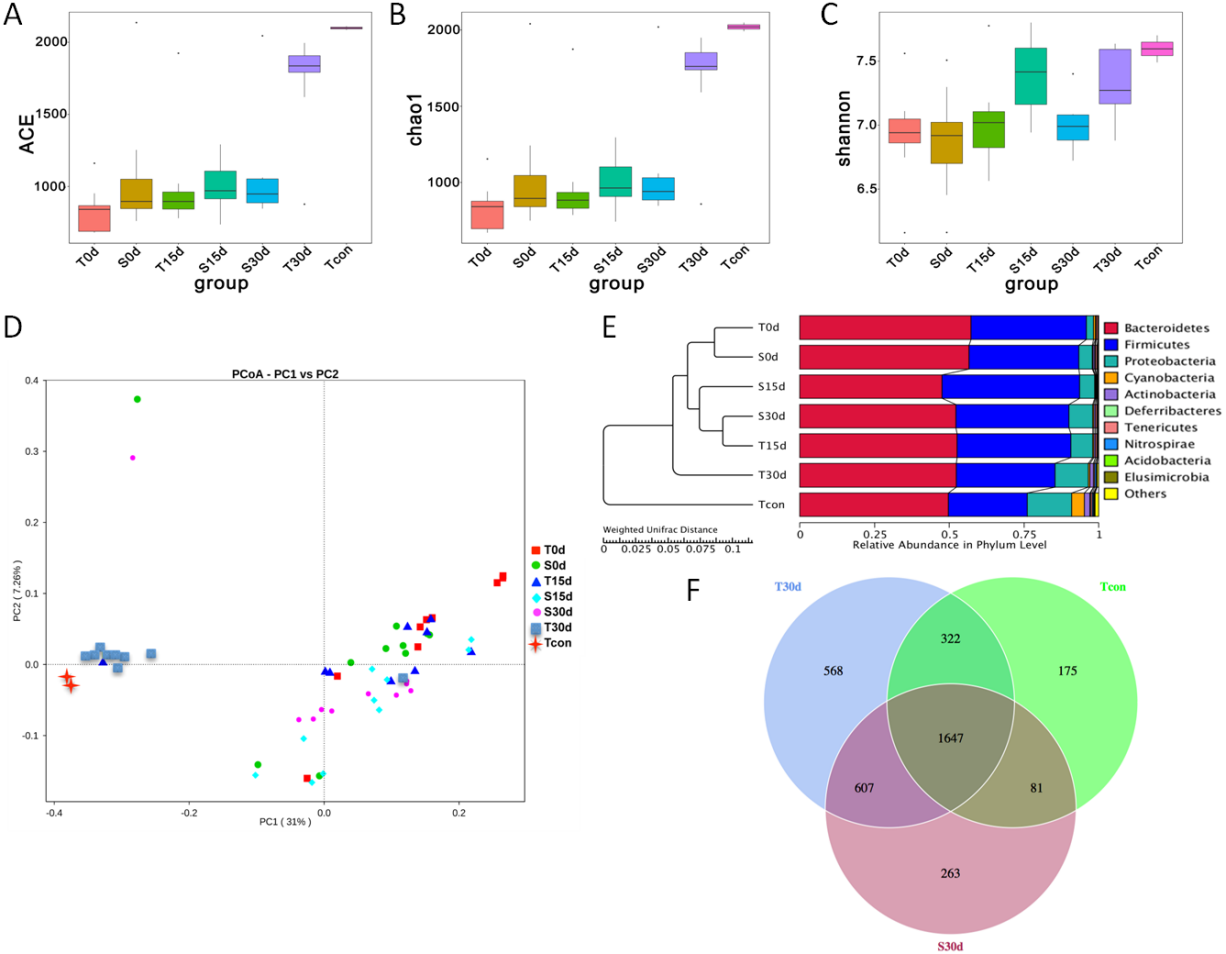
Comparison gut microbiota in experiment group and control group after FMT. (A) Results for the ACE index, where the value of the estimated number of OTUs in the community is proportional to the total number of species. (B) Results for the Chao1 index, where the estimated number of OTUs in the community is proportional to the total number of species. (C) Results of the Shannon diversity index, where the box plot represents the richness estimator. Significant differences are defined at the 95% confidence level. (D) PCoA unifrac plot based on the unweighted distance matrices between samples. Scatter plot showing principal coordinate 1 (PC1) vs principal coordinate 2 (PC2). The percentages represent the variation explained by the components. Each point represents a sample, and points with the same color come from the same group. The closer the distance between two points, the smaller the difference in composition of the two communities. The community composition for the Tcon and T30d samples clustered separately from the other samples and showed a different relative abundance pattern. (E) UPGMA cluster tree based on the unweighted Unifrac distance. The dendrogram shows the complete linkage hierarchical clustering of the T0d, T15d, T30d and S0d, S15d, S30d samples, and the bar charts show the relative abundance level of the bacterial phyla in each group. (F) Venn diagram of the T30d, S30d and Tcon samples (*n* = 9–10 mice per group).

To assess whether the composition of the gut microbiota in the experimental group shifted toward that of the guinea pig gut microbiota, further analyses were performed. The α-diversity analyses used were ACE (Fig. 3A) and Chao1 (Fig. 3B), both of which indicated that community richness for Tcon differed from that of the other samples, though the T30d values were more similar to Tcon values. These results suggest that the community richness of the control group gut microbiota remained stable, while that of the experimental group gut microbiota changed over time, especially by day 30 (T30d). The Shannon diversity index also indicated that there were differences in the gut microbial communities between samples. The Shannon diversity index value for T30d was significantly higher than those for T0d and T15d (Fig. 3C). The PCoA results for the bacterial communities in each sample revealed a decomposition pattern in two-dimensional space for the unweighted unifrac distances. According to the unweighted unifrac PCoA for community composition, Tcon and T30d clustered separately from the other groups and showed different relative abundance patterns (Fig. 3D). Our analysis of the relative abundance of bacteria at the phylum level in each sample showed that the values for T30d and Tcon were similar, and included more Proteobacteria and fewer Firmicutes than the other samples. The UPGMA (unweighted pair group method using arithmetic averages) cluster tree and relative abundance at the bacterial phylum level indicated that the floral structures of the Tcon and T30d samples showed greater similarity than those of the other samples (Fig. 3E). A Venn diagram shows the OTUs common and specific to T30d, S30d and Tcon (Fig. 3F). In addition to the 1647 OTUs common to T30d, S30d and Tcon, 322 OTUs were common to T30d and Tcon, and only 81 were common to S30d and Tcon. These results indicate that the composition of the gut microbiota in the experimental group had shifted towards that of the gut microbiota of the guinea pigs.

**Figure 4:**
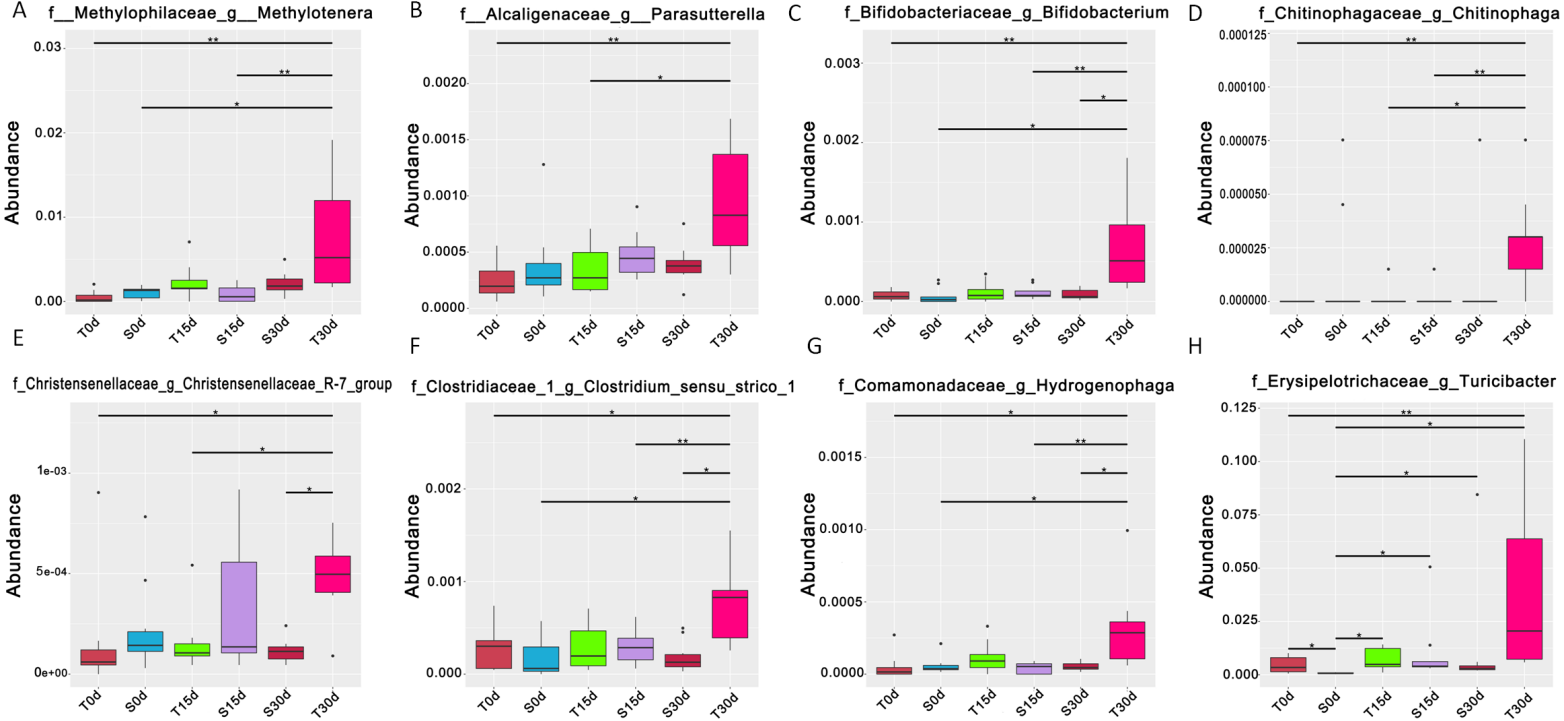
MetaStat analysis showing the bacterial abundance at the genus level across the different groups. Eight bacterial genera were significantly increased in T30d compared with T0d (*P* < 0.05 when compared T30d with T0d).

MetaStat analysis revealed that there was a change in the abundance of taxa at the genus level between samples (Fig. 4 and Fig. S3). The abundance of *Methylotenera*, *Parasutterella*, *Bifidobacterium*, *Chitinophaga*, *Christensenellaceae R-7 group*, *Clostridium sensu stricto 1*, *Hydrogenophaga* and *Turicibacter* was increased in T30d compared with the other samples (Fig. 4). Taken together, all of the above results indicate that after FMT with the guinea pig stool suspension for 30 days, the gut microbiota of mice had similar levels of bacterial richness and diversity as the gut microbiota of guinea pigs.

**Figure 5:**
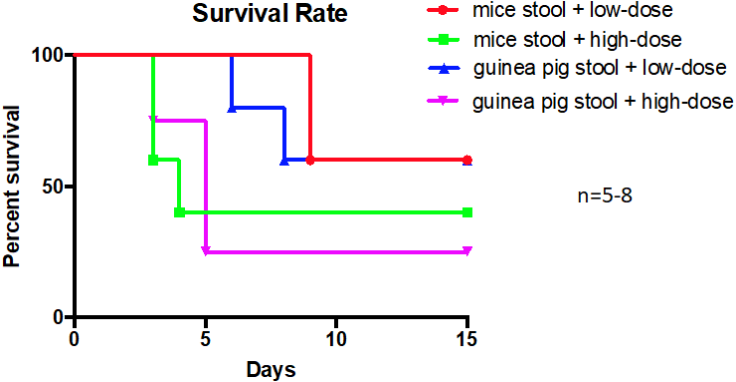
Survival rate curve. Mice were infected with *Yersinia pestis* after a 30-day guinea pig stool or mouse stool suspension transplant. The low dose group was injected with two colony forming units (CFU) of bacteria, and the high dose group was injected with fifteen CFU of bacteria. Days are shown in accordance with the abscissa and the ordinate percent survival to produce the survival curve. Control group (mouse diet and mouse stool suspension gavage): low dose shown in red; high dose shown in green. Experimental group (guinea pig diet and guinea pig stool suspension gavage): low dose shown in blue; high dose shown in purple. (log-rank test; *n* = 5–8 mice per group).

To observe whether these changes in the gut microbiota of mice administered a guinea pig stool FMT would affect their susceptibility to pneumonic plague, the mice were infected intranasally with *Y. pestis*. Experimental and control group mice were randomly divided into two groups, one of which was infected with a high dose of *Y. pestis* (15 CFU) and the other with a low dose of *Y. pestis* (2 CFU). The mice were then observed for 15 days, at which time no differences in survival rate were observed between groups (Fig. 5). The pathological sections of lung tissue harvested immediately after the mice were euthanized showed significantly effusion, edema and abscess (Fig. S4); however, no differences were observed between the experimental and control groups. This result suggests that even though the guinea pig gut microbiota had partially colonized in the mouse gut, it did not affect the susceptibility of the mice to pneumonic plague.

## Discussion

In addition to the three well-known biovars of *Y. pestis*, which are thought to be responsible for the three major plague pandemics (Perry & Fetherston, 1997), a fourth biovar, named microtus, has been identified and sequenced in our laboratory. Microtus is considered highly attenuated in guinea pigs, rabbits and humans, but is lethal in mice (Fan et al., 1995). Compared with the other *Y. pestis* biovars, microtus strains have a unique genomic profile highlighted by gene loss and pseudogene distribution. However, the specific loss of genes or gene functions documented for this group of strains is not completely responsible its attenuation in humans. The intestinal microbiome has been established to play a protective role during *S. pneumoniae* infection (Schuijt et al., 2016). Here, we investigated the possible role of gut microbiota in mice challenged with *Y. pestis* (microtus) pneumonic plague.

The composition of the gut microbiota differs between mice and guinea pigs (Fig. 3D), in part because mice are omnivores and guinea pigs are herbivores. In this study, the mouse gut was colonized by guinea pig bacteria by feeding the mice with a guinea pig diet for two months and then administering a guinea pig stool suspension daily by oral gavage for 30 days. Bacterial community profiles were evaluated by 16S rRNA gene sequencing, and the results showed that the community richness of these mice had increased after 30 days of guinea pig FMT (Figs. 2A–2C). The gut microbiota of these mice was more similar to that of the guinea pigs, indicating that the mouse intestinal tract had been partly colonized by guinea pig gut microbiota. However, when challenged with *Y. pestis* infection, these mice showed no improvement in survival rate compared with control mice, who had been fed a mouse diet and received a mouse fecal microbial transplantation. Thus, we did not observe that the guinea pig gut microbiota played a protective role in mice in the host defense against pneumonic plague.

To summarize, although the guinea pig intestinal microbiota was able to colonize the mouse intestinal tract, the susceptibility of these mice to pneumonic plague remained unchanged. One possible reason for this is that the intestinal microbiome has undergone coevolution with its host for trillions of years (Moeller et al., 2016) and the role of this microbial population in regulating the mouse immune system has also evolved over a very long time. Thus, guinea pig intestinal microbiota may not be equipped to regulate immune function in mice. Importantly, reported findings that plague resistance can be transmitted by fecal transplantation were based on transmission between members of the same species (*Meriones* rodents) (Assmar et al., 2018). Conversely, our research involved transmission to a different species (guinea pigs to mice). The other possible reason is that intestinal microbiota of healthy guinea pigs mainly composed of beneficial commensals and few pathobionts and pro-inflammatory organisms, which interact and restrict with each other, remain a relative homeostasis. Therefore, fecal microbial transplantation from guinea pigs to mice may not be able to alter the susceptibility of mice to pneumonic plague.

## Conclusions

*Y. pestis* strain 201 displays strong virulence in mice but not in guinea pigs, however, transplanted successfully the intestinal microbiota of guinea pigs to pneumonic plague-sensitive mice not affects mouse susceptibility to *Y. pestis* infection. We did not observe that the guinea pig gut microbiota played a protective role in mice defense against pneumonic plague.

##  Supplemental Information

10.7717/peerj.5637/supp-1 Figure S1 Bacterial changed obviously from day 0 to day 30 at group of guinea pig stool FMT The solid line is the relative abundance average. The dashed line is the median relative abundance. Each column represents the relative abundance of each sample in each group.

10.7717/peerj.5637/supp-2 Figure S2 Bacterial changed obviously from day 0 to day 30 at group of mice stool FMT The solid line is the relative abundance average. The dashed line is the median relative abundance. Each column represents the relative abundance of each sample in each group.

10.7717/peerj.5637/supp-3 Figure S3 MetaStat analysis showing the bacterial abundance at the genus level across the different groups

10.7717/peerj.5637/supp-4 Figure S4 The lung pathological observation from dead mice The low dose control group (mouse stool gavage) is shown in A, the low dose experimental group (guinea pig stool gavage) is shown in B, the high dose control group (mouse stool gavage) is shown in C, and the high dose experimental group (guinea pig stool gavage) is shown in D.

## Additional Information and Declarations

### DNA Deposition

The following information was supplied regarding the deposition of DNA sequences: NCBI BioProject ID: PRJNA437406.

### Data Availability

The following information was supplied regarding data availability: The raw data are provided in the Supplemental Files.
