# Supplementary figures and images for: Successful transplantation of guinea pig gut microbiota in mice and its effect on pneumonic plague sensitivity
Source: PeerJ. 2018 Sep 24;6:e5637. doi: 10.7717/peerj.5637 (PMC6160821; doi:10.7717/peerj.5637)

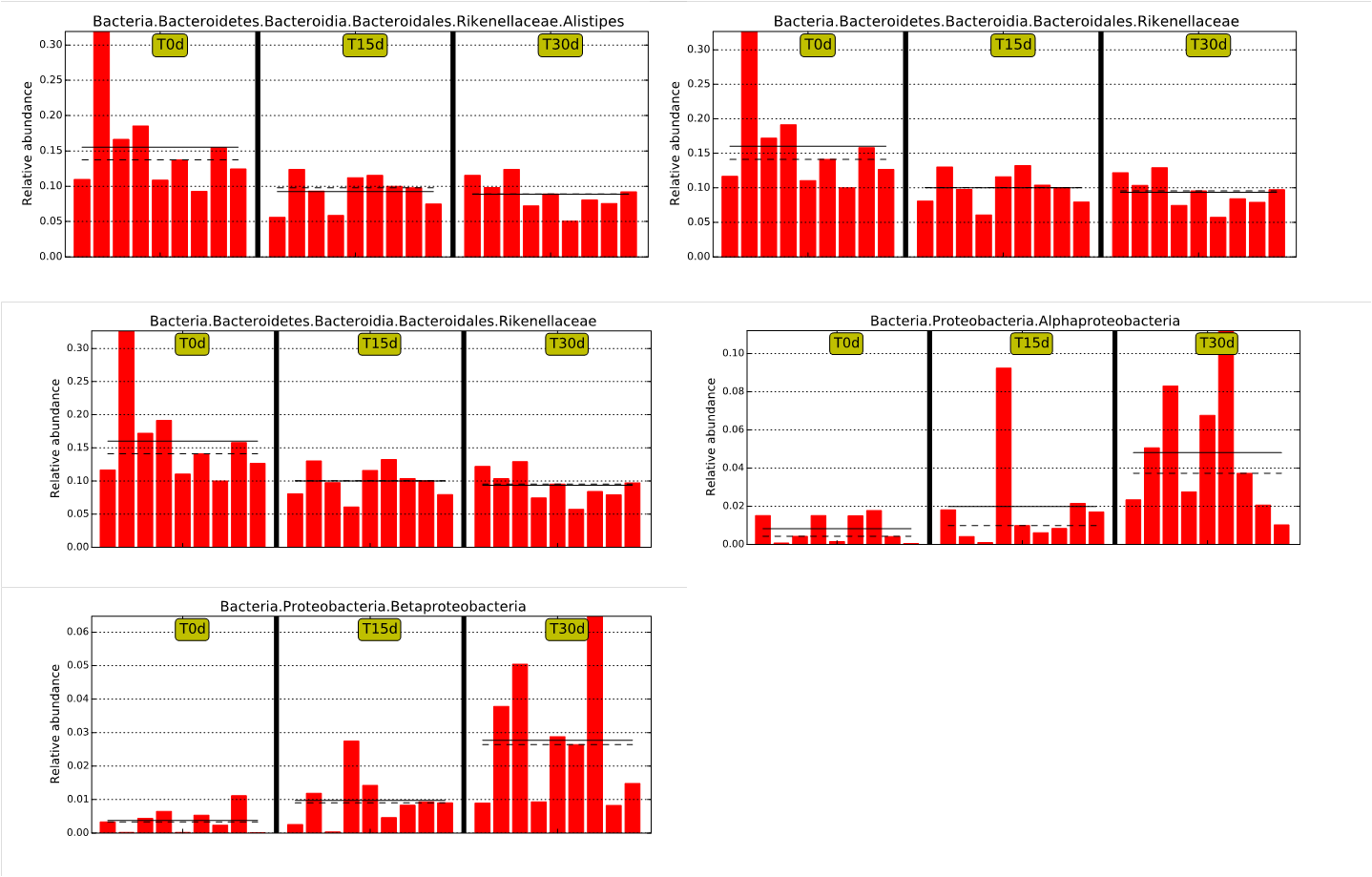

Supplement: Figure S1 — The solid line is the relative abundance average. The dashed line is the median relative abundance. Each column represents the relative abundance of each sample in each group. [file peerj-06-5637-s001.png]

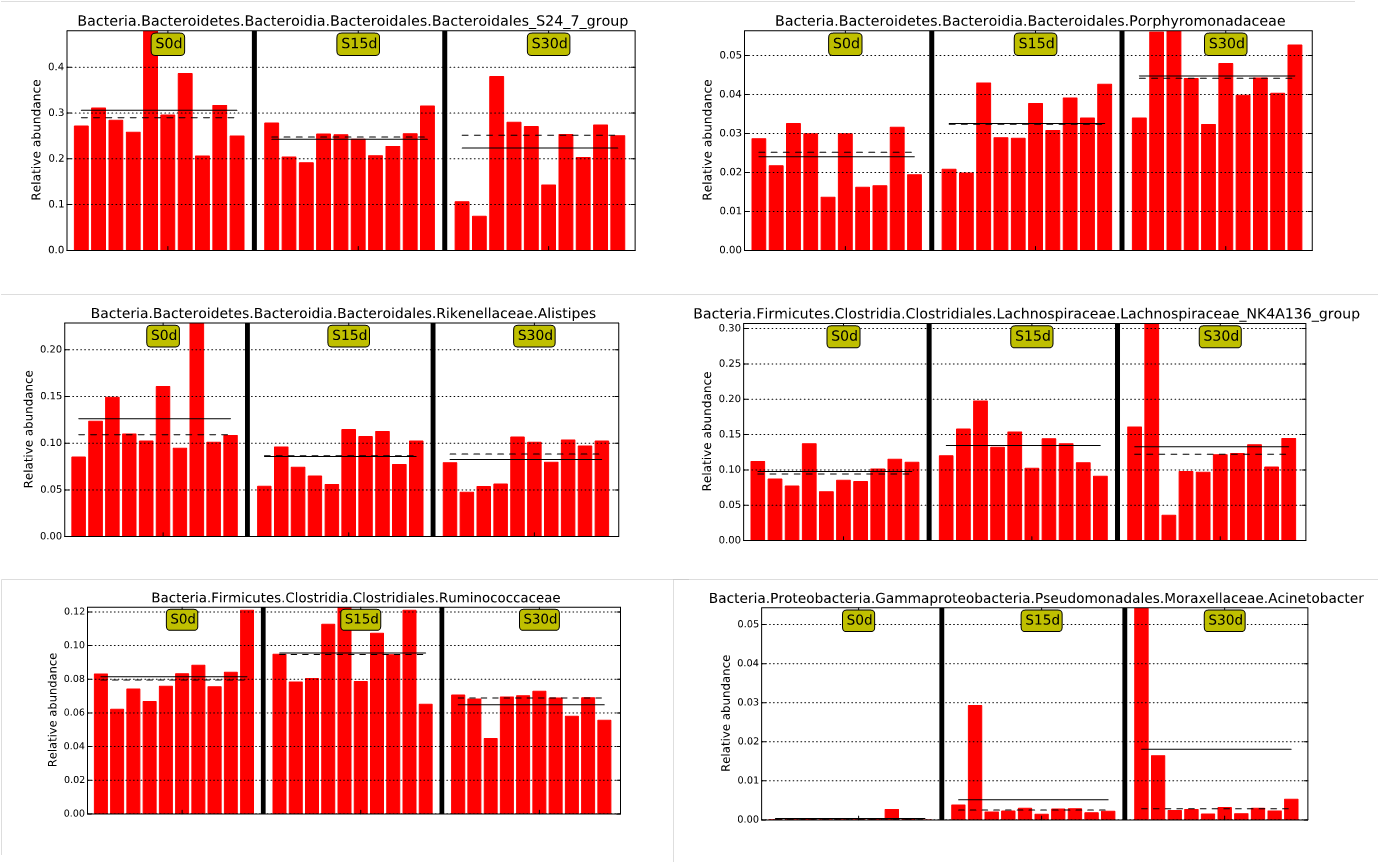

Supplement: Figure S2 — The solid line is the relative abundance average. The dashed line is the median relative abundance. Each column represents the relative abundance of each sample in each group. [file peerj-06-5637-s002.png]

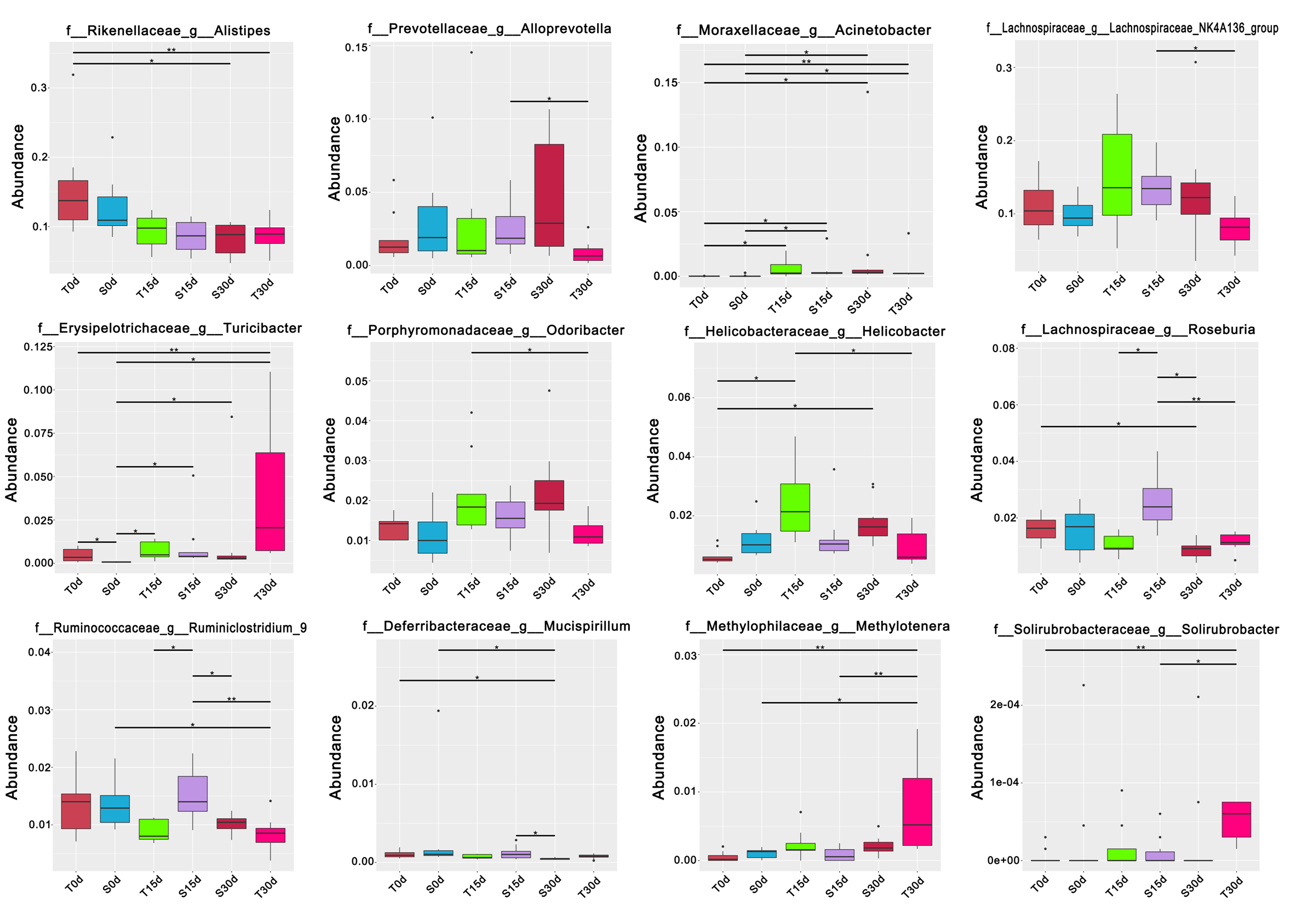

Supplement: Figure S3 [file peerj-06-5637-s003.png]

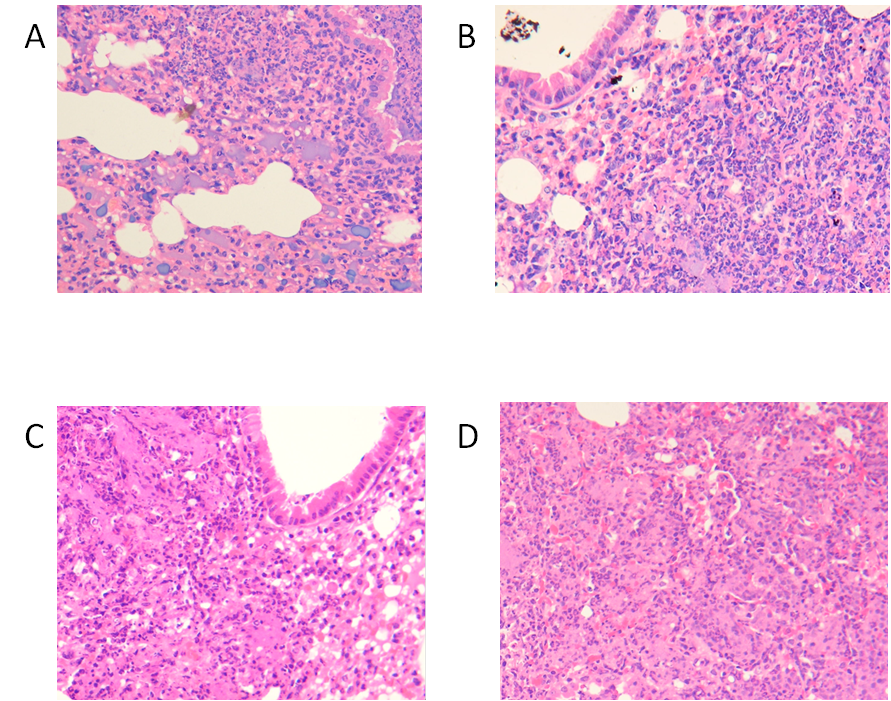

Supplement: Figure S4 — The low dose control group (mouse stool gavage) is shown in A, the low dose experimental group (guinea pig stool gavage) is shown in B, the high dose control group (mouse stool gavage) is shown in C, and the high dose experimental group (guinea pig stool gavage) is shown in D. [file peerj-06-5637-s004.png]
